# Targeting Methylglyoxal Metabolism to Enhance Ferroptosis Sensitivity in Tumor Therapy

**DOI:** 10.1002/advs.202505356

**Published:** 2025-09-04

**Authors:** Xinyue Zhang, Leng Han, Zimu Wang, Hanghui Yu, Jiao Liu, Rui Kang, Daolin Tang, Zhengjia Liu, Xianlong Du, Enyong Dai

**Affiliations:** ^1^ Second Division of Department of Oncology China‐Japan Union Hospital of Jilin University Changchun Jilin 130031 China; ^2^ DAMP Laboratory Department of Critical Care Medicine State Key Laboratory of Respiratory Disease The Third Affiliated Hospital Guangzhou Medical University Guangzhou Guangdong 510150 China; ^3^ Department of Surgery UT Southwestern Medical Center Dallas TX 75390 USA; ^4^ Department of thoracic surgery China‐Japan Union Hospital of Jilin University Changchun Jilin 130031 China; ^5^ Department of General Surgery, Peking Union Medical College Hospital Chinese Academy of Medical Science and Peking Union Medical College Beijing 100730 China

**Keywords:** autophagy, degradation, ferroptosis, methylglyoxal, pancreatic cancer

## Abstract

Ferroptosis, characterized by iron‐dependent lipid peroxidation, is a form of oxidative cell death increasingly recognized for its role in cancer therapy. The susceptibility of cancer cells to ferroptosis varies, highlighting the need to elucidate its underlying metabolic mechanisms. This study identifies a novel pathway in which the E3 ubiquitin ligase, praja ring finger ubiquitin ligase 1 (PJA1), mediates the proteasomal degradation of glyoxalase I (GLO1) exclusively in ferroptosis‐sensitive cancer cells. This degradation pathway is absent in ferroptosis‐resistant cells, resulting in differing management of methylglyoxal (MGO). The accumulation of MGO, as opposed to its clearance, facilitates ferroptosis by promoting the autophagic degradation of key anti‐ferroptotic proteins, specifically ferritin and glutathione peroxidase 4 (GPX4). Targeting the PJA1‐GLO1 axis through genetic and pharmacological means enhances the sensitivity of tumors to ferroptosis inducers across various preclinical models, including xenografts, orthotopic, and patient‐derived models. Additionally, clinical data demonstrate that elevated GLO1 expression is associated with poorer survival outcomes in pancreatic cancer patients. These findings suggest that modulating the MGO metabolism pathway, particularly through targeting the PJA1‐GLO1 axis, can amplify the effectiveness of ferroptosis‐inducing agents in cancer therapy.

## Graphical abstract

This study reveals how blocking a detox enzyme called GLO1 increases the buildup of a toxic molecule, methylglyoxal (MGO), which triggers cancer cell death by ferroptosis. A protein called PJA1 drives this process. Targeting the PJA1–GLO1 pathway boosts the effectiveness of ferroptosis‐inducing treatments in tumors, offering a promising approach for cancer therapy.

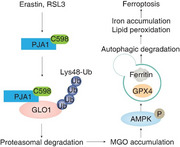

## 1 Introduction

Ferroptosis is a form of regulated cell death distinguished by an iron‐dependent accumulation of lipid peroxides that leads to oxidative damage within cells.^[^ ^1^, ^2^, ^3^ ^]^ Polyunsaturated fatty acids (PUFAs), owing to their multiple double bonds between carbon atoms, are particularly prone to peroxidation. These double bonds are susceptible to attacks by reactive oxygen species (ROS), culminating in lipid peroxidation—a critical mechanism in the initiation of ferroptosis.^[^ ^4^, ^5^ ^]^ Despite the presence of various antioxidant systems that maintain cellular redox homeostasis, glutathione peroxidase 4 (GPX4) is the principal enzymatic defense against lipid peroxidation.^[^ ^6^ ^]^ GPX4 utilizes glutathione (GSH) as a cofactor to reduce lipid hydroperoxides into their corresponding non‐toxic alcohol forms, thus inhibiting ferroptosis.^[^ ^7^ ^]^ Conversely, reduced GPX4 activity or GSH depletion can trigger ferroptosis via compounds such as RSL3 and erastin.^[^ ^8^ ^]^ Pharmacological induction of ferroptosis is increasingly recognized as a promising anticancer strategy, either as monotherapy or in combination with conventional chemotherapy, radiotherapy, or immunotherapy.^[^ ^9^ ^]^ Nevertheless, many cancer cells adapt their metabolism to enhance survival and confer resistance to cell death,^[^ ^10^ ^]^ highlighting the critical need to identify metabolic vulnerabilities when employing ferroptosis‐based therapies.^[^ ^11^, ^12^, ^13^, ^14^, ^15^, ^16^ ^]^

Methylglyoxal (MGO) is predominantly generated as a byproduct of glycolysis, through the breakdown of triose phosphates, including dihydroxyacetone phosphate and glyceraldehyde‐3‐phosphate.^[^ ^17^ ^]^ The primary detoxification pathway for MGO involves the glyoxalase system, consisting of glyoxalase I (GLO1) and glyoxalase II (GLO2).^[^ ^17^ ^]^ GLO1 catalyzes the conversion of MGO to S‐D‐lactoyl‐glutathione using GSH as a cofactor. GLO2 then hydrolyzes S‐D‐lactoyl‐glutathione to produce D‐lactate, regenerating GSH in the process. Increased MGO levels can lead to cellular toxicity and have been implicated in inflammation and aging‐related diseases.^[^ ^17^ ^]^ However, the impact of MGO metabolism on ferroptosis sensitivity remains poorly understood.

In this study, we reveal a novel pathway for GLO1 degradation in ferroptosis‐sensitive cancer cells, unlike in resistant cells, via the ubiquitin‐proteasome system (UPS) driven by praja ring finger ubiquitin ligase 1 (PJA1). This mechanism promotes MGO accumulation, which in turn triggers ferroptosis by facilitating the autophagic breakdown of GPX4 and ferritin. Our animal experiments show that both genetic and pharmacological inhibition of the PJA1‐GLO1 pathway amplifies the efficacy of ferroptosis‐targeted therapies in xenograft, orthotopic, and patient‐derived tumor models. Additionally, we demonstrate a clinical‐pathological correlation between high GLO1 expression and reduced survival in pancreatic cancer patients.

## 2 Results

### 2.1 MGO Levels Correlate with Ferroptosis Sensitivity

**Figure 1:**
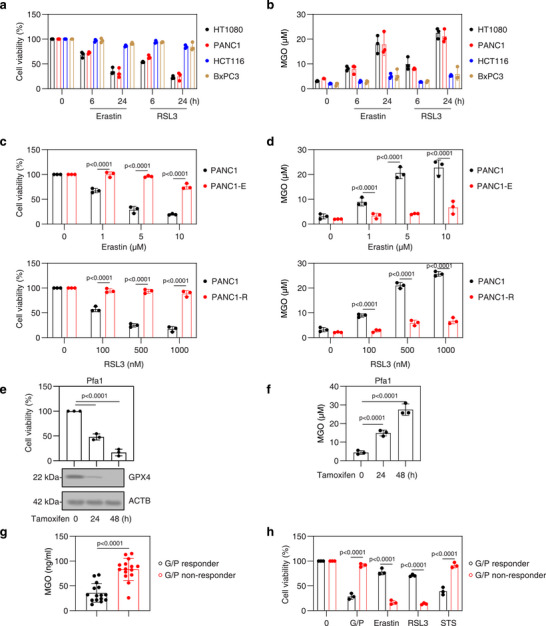
MGO levels correlate with ferroptosis sensitivity. a,b) Cell viability and intracellular MGO concentration in the indicated cancer cells treated with erastin (5 µm) or RSL3 (500 nm) for 6–24 h. c,d) Cell viability and intracellular MGO concentration in parental, erastin‐resistant PANC1 (PANC1‐E), and RSL3‐resistant PANC1 (PANC1‐R) cells treated with erastin (1–10 µm) or RSL3 (100–1000 nm) for 24 h. e,f) Analysis of GPX4 protein expression, cell viability, and intracellular MGO levels over a 0–48 h time course in Pfa1 cells following tamoxifen‐induced knockout of *Gpx4*. g) Serum MGO levels in PDAC patients categorized as G/P responders and non‐responders (n=15 cases per group). h) Primary PDAC cells from G/P responders and non‐responders were treated with G/P (100 nm), erastin (5 µm), RSL3 (500 nm), or staurosporine (STS, 500 nm) for 24 h. All semi‐quantitative data are presented as means ± SD; n = 3 biologically independent samples (a–f,h). Statistical analysis was performed using ANOVA with Tukey's multiple comparisons test (a‐f,h) or a t‐test (g).

To investigate the relationship between intracellular MGO concentration and sensitivity to ferroptosis, we measured MGO levels in cell lines previously characterized for their ferroptosis sensitivity (e.g., HT1080 and PANC1) and resistance (e.g., HCT116 and BxPC3).^[^ ^1^, ^6^, ^18^, ^19^ ^]^ Cell viability assays demonstrated that HT1080 and PANC1 cells exhibited greater sensitivity to identical concentrations of erastin or RSL3 treatment compared to HCT116 and BxPC3 cells (**Figure** **1**a). Quantitative assays revealed that HT1080 and PANC1 cells exhibited higher MGO concentrations compared to HCT116 and BxPC3 cells following treatment with erastin or RSL3 (Figure 1b). This suggests that cells intrinsically resistant to ferroptosis have lower MGO levels.

We subsequently developed erastin‐ or RSL3‐resistant derivatives of PANC1 cells (denoted PANC1‐E and PANC1‐R, respectively) through gradual drug exposure over 2 months.^[^ ^20^ ^]^ These acquired resistant cancer cell lines exhibited reduced sensitivity to erastin or RSL3 and demonstrated lower MGO concentrations compared to their parental counterparts (Figure 1c,d).

In addition to chemical inducers such as erastin and RSL3, we further validated this relationship using a tamoxifen‐inducible *Gpx4* knockout model (Pfa1 cells). *Gpx4* depletion led to increased MGO levels, reinforcing the link between ferroptotic stress and MGO accumulation (Figure 1e,f).

The standard combination therapy of gemcitabine and nab‐paclitaxel (G/P) in advanced PDAC has shown an overall response rate of only 23% in the Metastatic Pancreatic Adenocarcinoma Clinical Trial, compared to 7% for gemcitabine alone, highlighting the challenge of drug resistance.^[^ ^21^ ^]^ Our findings indicate that serum MGO levels are higher in the G/P‐nonresponse groups compared to those in the response groups (Figure 1g), suggesting that serum MGO could serve as a biomarker to predict G/P responsiveness. Moreover, primary PDAC cells from G/P‐nonresponse groups with elevated serum MGO levels showed increased sensitivity to the ferroptosis inducers erastin and RSL3, but not to the apoptosis inducer staurosporine (STS) (Figure 1h). In contrast, primary PDAC cells from the G/P‐response group displayed increased sensitivity to STS (Figure 1h). Therefore, targeting ferroptosis in the MGO‐high group could effectively target cells resistant to apoptosis in G/P‐nonresponse groups.

Taken together, these results suggest a negative correlation between MGO levels and both intrinsic and acquired resistance to ferroptosis in various cell lines and primary cancer cells.

### 2.2 The Proteasomal Degradation of GLO1 Mediated by PJA1 Promotes Ferroptosis

**Figure 2:**
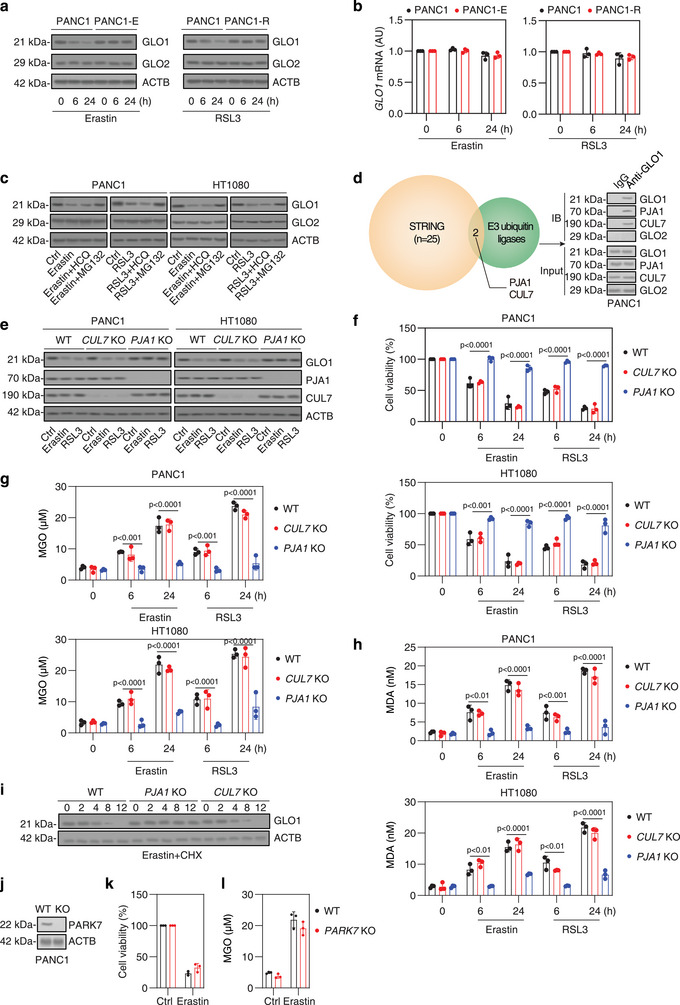
Proteasomal degradation of GLO1 mediated by PJA1 promotes ferroptosis. a,b) Analysis of GLO1 protein and mRNA expression in the indicated PANC1 cells treated with erastin (5 µm) or RSL3 (500 nm) for 6–24 h. c) Western blot analysis of protein expression in PANC1 and HT1080 cells after treatment with erastin (5 µm) or RSL3 (500 nm) for 24 h, with or without MG132 (5 µm) or HCQ (10 µm). d) Bioinformatic analysis using the STRING database identified PJA1 and CUL7 as potential E3 ligases interacting with GLO1. These interactions were experimentally validated in PANC1 cells by immunoprecipitation (IP) assays. e) Western blot analysis of GLO1 protein expression in the indicated PANC1 and HT1080 cells treated with erastin (5 µm) or RSL3 (500 nm) for 24 h. f–h) Analysis of cell viability, intracellular MGO and MDA concentrations in the indicated PANC1 and HT1080 cells treated with erastin (5 µm) or RSL3 (500 nm) for 6–24 h. i) Indicated PANC1 cells were treated with erastin (5 µM) and cycloheximide (CHX, 100 µg mL^−1^) for the indicated times (0–12 h). Whole‐cell lysates were subjected to immunoblotting for GLO1 and ACTB (loading control). j–l) The effects of *PARK7* knockout on erastin (5 µm)‐induced growth inhibition and MGO accumulation after 24 h in PANC1 cells. All semi‐quantitative data are presented as means ± SD for n = 3 biologically independent samples (b,f,g,h,k,l). Statistical analyses were conducted using ANOVA with Tukey's multiple comparisons test (f–h).

We used PANC1‐E and PANC1‐R, along with their parental counterparts, to explore potential alterations in the glyoxalase pathway, the primary defense against MGO toxicity. Western blot assays revealed that GLO1 protein levels were reduced by erastin or RSL3 in parental cells but remained unchanged in PANC1‐E and PANC1‐R cells (**Figure** **2**a). In contrast, GLO2 protein levels were stable across all cell lines (Figure 2a). Additionally, qPCR assays detected no significant changes in *GLO1* mRNA levels among these cell lines, with or without erastin or RSL3 treatment (Figure 2b), suggesting that transcriptional mechanisms were likely not involved.

The UPS and autophagy are two protein degradation pathways in mammalian cells that can regulate cell death sensitivity, including ferroptosis.^[^ ^22^, ^23^ ^]^ To determine which degradation pathway is involved in GLO1 protein degradation, we treated cells with MG132 and hydroxychloroquine (HCQ) to inhibit the UPS and autophagy, respectively. Western blot assays revealed that MG132, but not HCQ, blocked erastin‐ or RSL3‐induced GLO1 protein degradation in PANC1 and HT1080 cells (Figure 2c).

E3 ligases play a central role in the specificity of protein degradation by recognizing specific protein substrates and facilitating their ubiquitination, thus targeting them for proteasomal degradation.^[^ ^24^ ^]^ Using a bioinformatics approach, we queried the STRING database (https://string‐db.org/) to identify potential E3 ligases that interact with GLO1. This analysis predicted PJA1 and Cullin 7 (CUL7) as candidate binding partners based on known and predicted protein–protein interactions (Figure 2d). Consistent with these predictions, immunoprecipitation assays confirmed that GLO1 physically interacts with both PJA1 and CUL7 in PANC1 cells (Figure 2D). CRISPR‐Cas9‐based knockout experiments demonstrated that only the absence of *PJA1*, and not *CUL7*, inhibited erastin‐ or RSL3‐induced GLO1 protein degradation and the associated sensitivity to ferroptosis in PANC1 and HT1080 cells (Figure 2e,f). The assay of MGO concentrations in these knockout cells confirmed that the degradation of GLO1 protein mediated by PJA1 is responsible for the accumulation of MGO during ferroptosis (Figure 2g). As expected, the knockout of *PJA1*, but not *CUL7*, inhibited the production of malondialdehyde (MDA), an end‐product of PUFA peroxidation,^[^ ^25^ ^]^ induced by erastin or RSL3 (Figure 2h). Cycloheximide (CHX) chase assay revealed that knockout of *PJA1*—but not *CUL7*—increased GLO1 protein stability in response to erastin treatment in PANC1 cells (Figure 2i).

A recent study reported that expression of parkinsonism associated deglycase (PARK7, also known as DJ‐1) in HEK293 cells conferred resistance to erastin‐induced ferroptosis, in part by preventing the accumulation of MGO.^[^ ^26^ ^]^ However, *PARK7* knockout did not alter erastin‐induced growth inhibition or MGO accumulation in PANC1 cells (Figure 2j–l).

Collectively, these findings indicate that the proteasomal degradation of GLO1, mediated by PJA1, contributes to MGO accumulation, lipid peroxidation, and ferroptosis in cancer cells.

### 2.3 Cysteine 598 of PJA1 is Essential for the Degradation of GLO1 and Subsequent Ferroptosis

**Figure 3:**
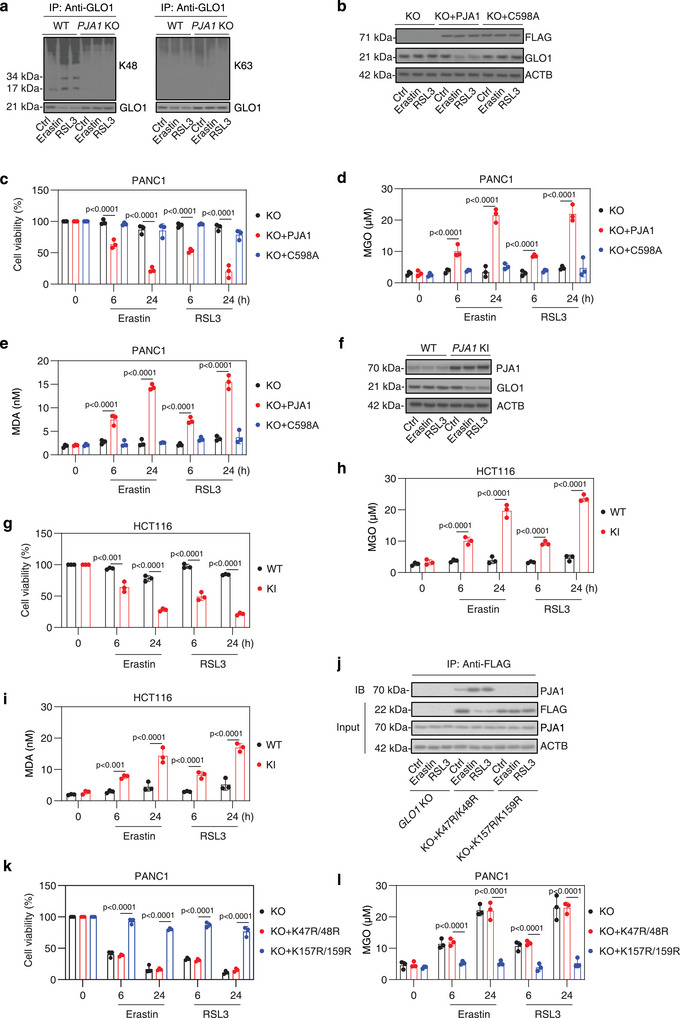
Cysteine 598 of PJA1 is required for the degradation of GLO1 and subsequent ferroptosis. a) Analysis of *PJA1* knockout (KO) on the binding of K48‐ and K63‐linked ubiquitination of GLO1 protein in the indicated PANC1 cells treated with erastin (5 µm) or RSL3 (500 nm) for 6 h. b) Western blot analysis of GLO1 protein expression in the indicated *PJA1*‐KO PANC1 cells with or without the expression of wild‐type (WT) or C598A mutant after treatment with erastin (5 µm) or RSL3 (500 nm) for 24 h. c–e) Analysis of cell viability, intracellular MGO, and MDA concentrations in the indicated PANC1 cells treated with erastin (5 µm) or RSL3 (500 nm) for 6–24 h. f) Western blot analysis of GLO1 protein expression in WT or *PJA1* overexpressed (KI) HCT116 cells after treatment with erastin (5 µm) or RSL3 (500 nm) for 24 h. g–i) Analysis of cell viability, intracellular MGO, and MDA concentrations in the indicated HCT116 cells treated with erastin (5 µm) or RSL3 (500 nm) for 6–24 h. j) Analysis of the interaction between GLO1 and PJA1, as well as GLO1 degradation, in *GLO1*‐KO PANC1 cells expressing either K47R/K48R or K157R/K159R GLO1 mutants, following treatment with erastin (5 µm) or RSL3 (500 nm) for 24 h. k,l) Analysis of cell viability and intracellular MGO in the indicated PANC1 cells treated with erastin (5 µm) or RSL3 (500 nm) for 6–24 h. All semi‐quantitative data are presented as means ± SD for n = 3 biologically independent samples (c–e,g–i,k,l). Statistical analyses were conducted using ANOVA with Tukey's multiple comparisons test (c–e,g–i,k,l).

Multiple ubiquitin molecules are attached to a target protein, forming a polyubiquitin chain. Our analysis revealed that the knockout of *PJA1* inhibited K48‐linked ubiquitination, but did not affect K63‐linked ubiquitination of GLO1 in PANC1 cells following treatment with erastin or RSL3 (**Figure** **3**a). The PJA1 protein contains a really interesting new gene (RING) finger domain, a zinc‐finger motif that is a characteristic feature of E3 ubiquitin ligases.^[^ ^27^ ^]^ This RING domain is crucial for facilitating the transfer of ubiquitin molecules from the E2 ubiquitin‐conjugating enzymes to substrate proteins, thereby enabling the formation of polyubiquitin chains that regulate protein function and degradation.

To assess the importance of the RING domain in PJA1's function, we introduced a C598A mutation into the RING finger domain of PJA1, which is known to disrupt its E3 ligase activity.^[^ ^28^ ^]^ Re‐expression of wild‐type (WT) PJA1, but not the C598A mutant, restored erastin‐ or RSL3‐induced GLO1 degradation in *PJA1*‐knockout (KO) PANC1 cells (Figure 3b). This finding highlights the critical role of the RING domain in PJA1's ability to promote K48‐linked ubiquitination and mediate GLO1 degradation.

Functionally, reintroducing WT PJA1, but not the C598A mutant, increased sensitivity to erastin or RSL3, which was associated with elevated MGO and MDA accumulation in *PJA1*‐knockout cells (Figure 3c–e). This suggests that PJA1's E3 ligase activity is essential for sensitizing cells to ferroptosis‐inducing agents. Moreover, overexpressing *PJA1* in relatively ferroptosis‐resistant HCT116 cell line not only enhanced GLO1 degradation (Figure 3f) and sensitivity to erastin and RSL3 (Figure 3g) but also led to increased accumulation of MGO and MDA (Figure 3h,i).

To identify the specific ubiquitination sites on GLO1 required for PJA1 recognition, we focused on lysine (K) residues, the canonical acceptor sites for ubiquitin. Human GLO1 (UniProtKB: Q04760) contains 18 lysines, and we employed a heuristic analysis based on lysine clustering and positional accessibility (e.g., proximity to N‐ or C‐termini) to predict potential ubiquitination sites.^[^ ^29^ ^]^ This approach identified K47/K48 and K157/K159 as top candidates (score = 2; Table S1, Supporting Information). Functional analysis using K→R point mutants revealed that mutation of K157/K159—but not K47/K48—disrupted GLO1 binding to PJA1 and attenuated GLO1 degradation, MGO accumulation, and ferroptosis sensitivity (Figure 3j–l). These results indicate that K157/K159 are critical sites for PJA1‐mediated ubiquitination and degradation of GLO1.

### 2.4 MGO Induces Autophagy‐Dependent Ferroptosis

**Figure 4:**
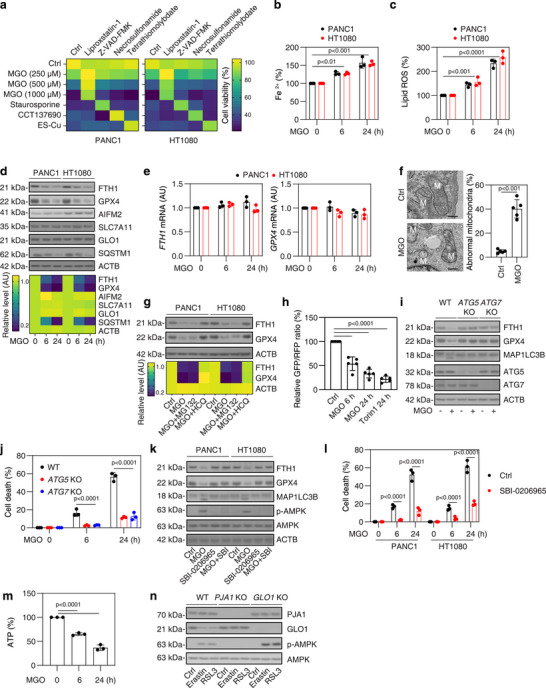
MGO induces autophagy‐dependent ferroptosis. a) Analysis of cell viability in PANC1 and HT1080 cells following treatment with MGO (250–1000 µm), staurosporine (500 nm), CCT137690 (5 µm), or ES‐Cu (200 nm) in the absence or presence of liproxstatin‐1 (1 µm), Z‐VAD‐FMK (5 µm), necrosulfonamide (1 µm), and tetrathiomolybdate (5 µm) for 24 h. b–e) Analysis of Fe^2^⁺, lipid ROS, and indicated protein and mRNA expression in PANC1 and HT1080 cells following treatment with MGO (500 µm) for 6–24 h. f) Transmission electron microscopy analysis of mitochondrial morphology in PANC1 cells treated with MGO (500 µm) for 24 h. g) Western blot analysis of indicated protein expression in PANC1 and HT1080 cells following treatment with MGO (500 µm) in the absence or presence of MG132 (5 µm) or HCQ (10 µm) for 24 h. h) Autophagic flux was assessed using a microplate reader in PANC1 cells expressing the GFP‐MAP1LC3–RFP‐MAP1LC3ΔG reporter. Cells were treated with MGO (500 µm) or Torin1 (1 µm) for the indicated durations. The GFP/RFP fluorescence ratio was calculated and expressed as a percentage relative to the baseline value at time 0. i,j) Analysis of protein expression and cell death in WT, *ATG5*‐KO, and A*TG7*‐KO PANC1 cells following treatment with MGO (500 µm) for 24 h. k,l) Analysis of protein expression and cell death in PANC1 and HT1080 cells following treatment with MGO (500 µm) in the absence or presence of SBI‐0206965 (1 µm) for 24 h. All semi‐quantitative data are presented as means ± SD for n = 3 or 5 biologically independent samples (b,c,e,f,h,j,l). Relative protein levels are presented as a heat map (d,g), with the untreated group normalized to a value of 1. Statistical analyses were conducted using ANOVA with Tukey's multiple comparisons test (b,c,h,j,l,m) or t test (f).

Several studies have demonstrated the detrimental effects of exogenous MGO in inducing apoptosis in endothelial cells.^[^ ^30^, ^31^, ^32^ ^]^ To determine whether MGO has a similar impact on cancer cells, we treated PANC1 and HT1080 cells with varying concentrations of exogenous MGO. The cytotoxic effects of MGO were reversed by the ferroptosis inhibitor liproxstatin‐1, but not by other cell death inhibitors, including Z‐VAD‐FMK (for apoptosis), necrosulfonamide (for necroptosis), and tetrathiomolybdate (for cuproptosis) (**Figure** **4**a). As controls, these inhibitors effectively blocked their respective inducers of cell death: staurosporine for apoptosis, CCT137690 for necroptosis,^[^ ^33^ ^]^ and ES‐Cu for cuproptosis (Figure 4a).^[^ ^34^, ^35^ ^]^

Subsequently, we investigated the impact of MGO on key events in ferroptosis, specifically iron accumulation and lipid peroxidation.^[^ ^3^ ^]^ Quantitative assays revealed that MGO increased Fe^2^⁺ levels and lipid ROS (Figure 4b,c). Western blot analysis further demonstrated that MGO decreased the protein levels of ferritin heavy chain 1 (FTH1) and GPX4, two key regulators of iron accumulation and lipid peroxidation during ferroptosis (Figure 4d). As a control, the autophagic substrate sequestosome 1 (SQSTM1) was also degraded by MGO (Figure 4D). In contrast, MGO treatment did not affect the mRNA levels of *FTH1* and *GPX4* (Figure 4e), nor did it alter the protein expression of other key ferroptosis regulators such as AIF family member 2 (AIFM2, also known as FSP1) and solute carrier family 7 member 11 (SLC7A11) (Figure 4d). Transmission electron microscopy analysis revealed characteristic ferroptotic changes, including increased membrane density and loss of mitochondrial cristae (Figure 4f). MGO stimulation did not change GLO1 protein expression (Figure 4d). Pharmacological inhibition of the UPS using MG132 and blockade of autophagy using HCQ revealed that the downregulation of FTH1 and GPX4 was inhibited by HCQ, but not by MG132 (Figure 4g), suggesting an autophagy‐dependent mechanism.

To further evaluate autophagic flux, we employed the GFP‐MAP1LC3–RFP‐MAP1LC3ΔG probe, which quantifies autophagic degradation by comparing the levels of GFP‐MAP1LC3 to the undegradable RFP‐MAP1LC3ΔG internal control.^[^ ^36^ ^]^ This assay revealed that MGO treatment reduced the GFP/RFP ratio (Figure 4h), consistent with an increase in autophagic flux. The mTOR inhibitor Torin1 was used as a positive control (Figure 4h).

To further confirm that MGO‐induced protein degradation of ferritin and GPX4 is dependent on autophagy, we utilized *ATG5*‐KO and *ATG7*‐KO PANC1 cells. Depletion of *ATG5* or *ATG7* inhibited the production of the autophagosome marker microtubule‐associated protein 1 light chain 3 beta (MAP1LC3B)‐II, whereas preserving the protein levels of FTH1 and GPX4 following MGO treatment (Figure 4i). Cell death assays further demonstrated that depletion of *ATG5* or *ATG7* attenuated MGO‐induced cytotoxicity (Figure 4j). These results suggest that MGO induces ATG5‐ and ATG7‐dependent autophagy and ferroptosis.

Next, we examined the effects of MGO on the activation of AMP‐activated protein kinase (AMPK), the kinase responsible for the initiation of autophagy and ferroptosis.^[^ ^37^, ^38^ ^]^ Indeed, MGO activated AMPK, as evidenced by assays measuring AMPK phosphorylation (Figure 4k). Furthermore, the potent AMPK inhibitor SBI‐0206965 prevented MGO‐induced formation of MAP1LC3B‐II (Figure 4k), as well as the degradation of FTH1 and GPX4 proteins (Figure 4k), and mitigated cell cytotoxicity (Figure 4l).

It is well established that MGO modifies mitochondrial proteins through glycation, leading to impaired oxidative phosphorylation and reduced ATP production.^[^ ^39^, ^40^ ^]^ As expected, MGO treatment resulted in ATP depletion (Figure 4m), a known trigger for AMPK activation.^[^ ^41^ ^]^ Moreover, *PJA1* knockout inhibited AMPK activation, whereas *GLO1* knockout enhanced AMPK activation in response to erastin or RSL3 (Figure 4n), suggesting that MGO accumulation contributes to metabolic stress and AMPK‐mediated energy sensing during ferroptosis.

Collectively, these findings demonstrate that MGO triggers the activation of AMPK, initiating ATG‐dependent autophagy for the degradation of ferritin and GPX4, which promotes ferroptosis.

### 2.5 Targeting MPO Pathway Enhances Ferroptosis Sensitivity In Vivo

**Figure 5:**
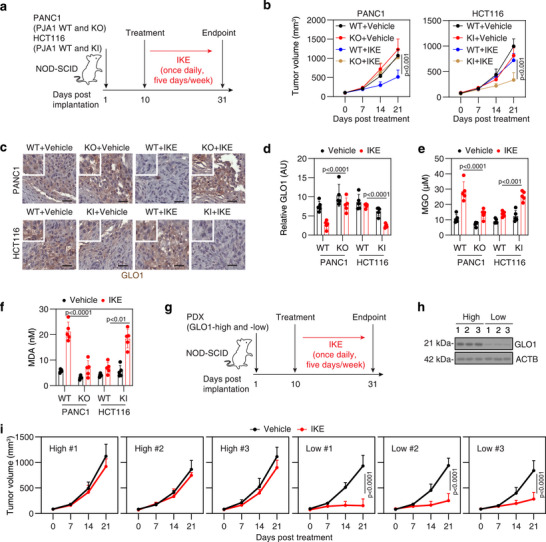
Targeting MPO pathway enhances ferroptosis sensitivity in xenograft models. a) Schematic of the experimental setup showing subcutaneous implantation of human PANC1 or HCT116 cells in immunocompromised NOD‐SCID mice, followed by a treatment protocol over 3 weeks. b) Growth curves for tumors from subcutaneously implanted the indicated PANC1 or HCT116 cells in NOD‐SCID mice (n = 5 per group) following the treatment described in (a). c–f) Analysis of GLO1, MGO, and MDA levels in isolated tumors on day 31 (n = 5 mice per group). Bar = 100 µm. g) Schematic of the experimental protocol for subcutaneous implantation of PDXs in NOD‐SCID mice, including a 3‐week treatment regimen. h) Western blot analysis showing GLO1 expression levels in PDAC tumors categorized by high or low expression. i) Tumor growth curves for PDXs subcutaneously implanted in NOD‐SCID mice (n = 5 per group). Data are presented as means ± SD, with statistical analysis performed using ANOVA (b,d–f,i).

Imidazole‐ketone‐erastin (IKE) is a metabolically stable inducer of ferroptosis that has been used in mouse models.^[^ ^42^ ^]^ To investigate the role of PJA1 in modulating ferroptosis sensitivity in vivo, we compared the response of WT and *PJA1*‐KO PANC1 cells in xenograft models in NOD‐SCID IL2rγ^null^ mice (**Figure** **5**a). The depletion of *PJA1* impaired the anticancer activity of IKE in PANC1 xenograft models (Figure 5b). In contrast, *PJA1*‐KI in HCT116 cells enhanced sensitivity to IKE in xenograft models (Figure 5b).

Immunohistochemical (IHC) staining revealed that GLO1 expression was elevated in *PJA1*‐KO PANC1 group, whereas it was reduced in *PJA1*‐KI HCT116 group compared to their respective controls following IKE treatment (Figure 5c). Quantitative assays measuring MGO and MDA concentrations in tumor tissues showed a negative correlation with GLO1 expression (Figure 5d–f). These results suggest that genetic modulation of PJA1 alters GLO1 protein expression, which in turn influences MGO levels and modulates sensitivity to ferroptosis inducers in immunodeficient mice.

We further examined the response to IKE in patient‐derived tumor models (PDX) in NOD‐SCID IL2rγ^null^ mice (Figure 5g). Pancreatic tumors with high GLO1 expression, obtained surgically, demonstrated diminished responsiveness to IKE compared to tumors with low GLO1 expression (Figure 5h,i). These findings further support the hypothesis that GLO1 serves as a negative regulator of ferroptosis sensitivity in vivo.

**Figure 6:**
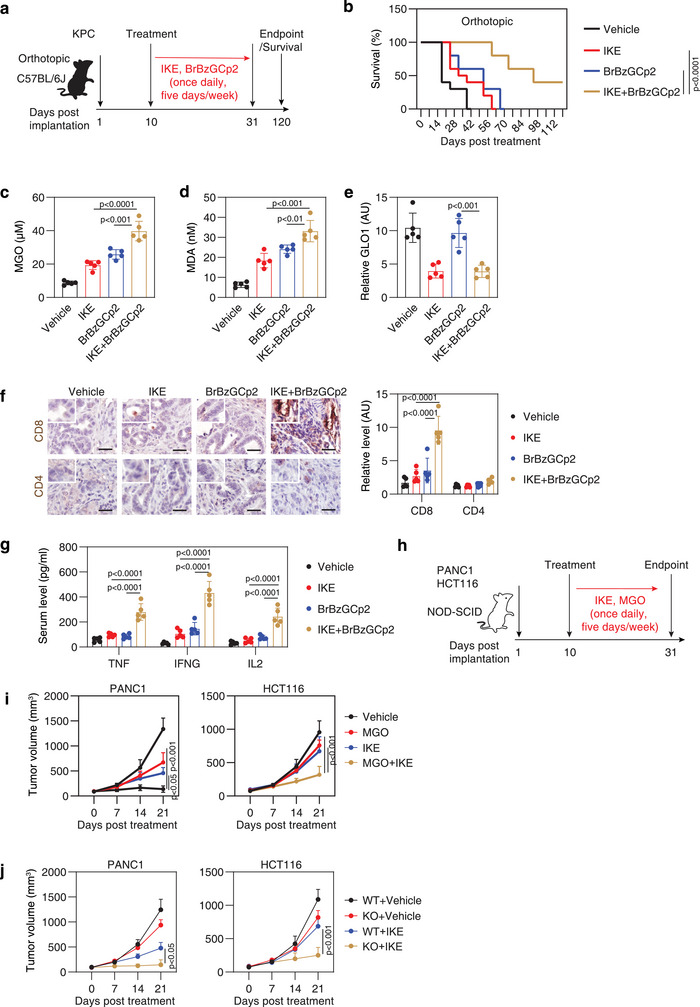
Targeting MPO pathway enhances ferroptosis sensitivity in orthotopic and xenograft models. a) Schematic diagram of the experimental setup: orthotopic implantation of mouse KPC cells in C57BL/6J mice, followed by a 3‐week treatment protocol. b) Survival curves for mice treated with vehicle, IKE, and/or BrBzGCp2 (n = 20 mice per group). c–e) Analysis of MGO, MDA, and GLO1 levels in isolated tumors on day 28 (n = 5 mice per group). f) IHC analysis of CD8 and CD4 in isolated tumors on day 28 (n = 5 mice per group). Bar = 100 µm. g) ELISA analysis of serum cytokines on day 28 (n = 5 mice per group). h) Schematic of the experimental setup showing subcutaneous implantation of human PANC1 or HCT116 cells in immunocompromised NOD‐SCID mice, followed by a treatment protocol over 3 weeks. i) Growth curves for tumors from subcutaneously implanted the indicated PANC1 or HCT116 cells in NOD‐SCID mice (n = 5 per group) following the treatment described in (h). j) Growth curves for tumors from subcutaneously implanted the indicated WT and *GLO1*‐KO PANC1 or HCT116 cells in NOD‐SCID mice (n = 5 per group) following the treatment with IKE. Data are presented as means ± SD. Statistical analyses were conducted using ANOVA for panels c–g, i, and j, with log‐rank tests for panel B.

Next, we investigated whether combining the GLO1 inhibitor BrBzGCp2 with IKE could enhance anticancer efficacy in an orthotopic model using immunocompetent C57BL/6J mice (**Figure** **6**a). The combination of BrBzGCp2 and IKE extended survival compared to either agent alone in C57BL/6J mice with orthotopically implanted KPC cells from pancreatic tumors of KPC mice (*Pdx1‐Cre;K‐Ras^G12D/+^;p53^R172H/+^*) (Figure 6b). In separate experiments, quantitative assays measuring MGO and MDA concentrations in tumors at day 28 post‐treatment revealed that the combination therapy elevated MGO levels and induced lipid peroxidation (Figure 6c,d). As expected, IKE inhibited GLO1 expression, whereas BrBzGCp2 had no effect on GLO1 expression (Figure 6e). IHC staining further revealed increased infiltration of CD8^+^ (but not CD4^+^) T cells in tumors from combination‐treated mice (Figure 6f). This process was associated with elevated serum cytokines linked to cytotoxic T cells, including tumor necrosis factor (TNF), interferon gamma (IFNG, also known as IFN‐γ), and interleukin 2 (IL2) (Figure 6g), suggesting that this treatment strategy not only enhances ferroptosis but also stimulates adaptive immune responses in immunocompetent mice.

We also evaluated whether oral administration of MGO could enhance the antitumor efficacy of IKE in xenograft models. Prior toxicological studies have shown that MGO is well tolerated in mice at doses up to 50 mg kg^−1^ per day via oral gavage.^[^ ^43^ ^]^ In line with this, co‐treatment with MGO and IKE suppressed tumor growth in xenografts established from PANC1 and HCT116 cells, compared to either agent alone (Figure 6h,i). Similarly, *GLO1* knockout potentiated the antitumor effect of IKE in both PANC1 and HCT116 xenografts (Figure 6j), further supporting the role of GLO1 in ferroptosis resistance and tumor progression.

### 2.6 Clinical Significance of PJA1 and GLO1 Expression in PDAC Patients

**Figure 7:**
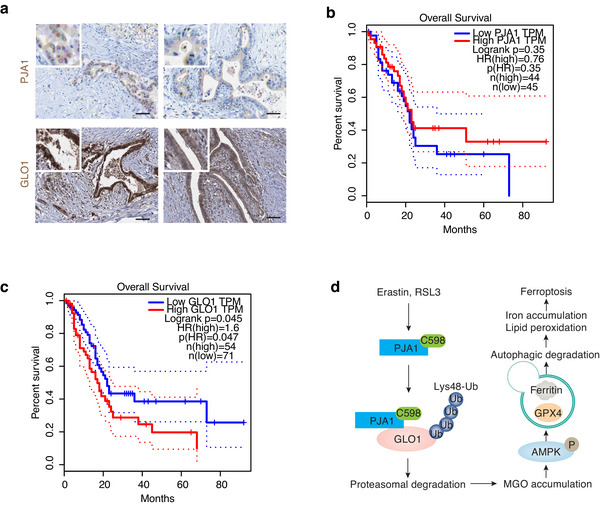
Clinical significance of PJA1 and GLO1 expression in PDAC patients. a) Analysis of PJA1 and GLO1 expression and localization in pancreatic tumors using The Human Protein Atlas. Bar = 100 µm. b,c) Correlation of PJA1 or GLO1 expression with survival in PDAC patients using The Cancer Genome Atlas. The selected cut‐off was the optimal choice. d) A model illustrating PJA1‐mediated GLO1 proteasomal degradation, leading to ferroptosis via MGO accumulation and subsequent autophagic degradation of ferritin and GPX4.

We first utilized The Human Protein Atlas (https://www.proteinatlas.org/) to assess PJA1 and GLO1 protein expression in PDAC patients. IHC staining revealed that PJA1 and GLO1 were predominantly expressed in the ductal cells of the pancreatic tumor (**Figure** **7**a). To assess the clinical relevance of PJA1 and GLO1 expression in PDAC, we analyzed mRNA expression data from The Cancer Genome Atlas (https://portal.gdc.cancer.gov/). Our analysis revealed that higher mRNA expression of *PJA1* was associated with a trend toward increased survival, although the P‐value did not reach statistical significance (P > 0.05, Figure 7b). Conversely, elevated *GLO1* mRNA expression was significantly correlated with reduced survival in PDAC patients (P < 0.05, Figure 7c). These results indicate that PJA1 and GLO1 may have distinct and potentially antagonistic roles in the progression and clinical outcomes of PDAC.

## 3 Discussion

The interplay between cellular metabolism and ferroptosis is complex, with disruptions in metabolic pathways influencing a cell's susceptibility or resistance to oxidative damage.^[^ ^44^ ^]^ This has implications for cancer therapy and other diseases associated with iron‐related damage.^[^ ^45^ ^]^ In this study, we report that increased MGO production contributes to ferroptosis in cancer cells (Figure 7d). Specifically, we identify that PJA1‐mediated GLO1 proteasomal degradation impairs the detoxification of MGO. Consequently, elevated MGO activates selective autophagy, leading to the degradation of key antiferroptotic proteins, including ferritin and GPX4, thereby increasing iron accumulation and lipid peroxidation. These findings offer mechanistic insights into how dysregulated metabolism and degradation pathways collectively modulate ferroptosis sensitivity.

MGO is highly reactive and can cause cellular damage by forming advanced glycation end‐products when it reacts with proteins, lipids, and nucleic acids.^[^ ^46^ ^]^ The GLO1‐GLO2 system preserves cellular homeostasis by preventing the accumulation of MGO and mitigating oxidative stress. Dysregulation of GLO1 expression or activity has been linked to various diseases, including diabetes, neurodegenerative diseases, and cancer, as elevated MGO levels lead to cellular toxicity and promote pathological processes, such as inflammation and fibrosis.^[^ ^46^ ^]^ In *Brassica napus* (rapeseed), research has shown that GLO1 degradation in the stigma contributes to a self‐incompatibility response, involving the proteasomal degradation of GLO1 by the E3 ubiquitin ligase armadillo repeat‐containing 1 (ARC1).^[^ ^47^ ^]^ In mammalian cells, itaconate, a product of the tricarboxylic acid cycle derived from citrate, drives pro‐inflammatory responses in macrophages through GLO1 proteasomal degradation via Cys139.^[^ ^48^ ^]^ In current study, we demonstrate that PJA1, but not CUL7, is the key E3 ligase responsible for the K48 ubiquitination of GLO1 and its subsequent degradation. PJA1 has been linked to the regulation of apoptosis by promoting forkhead box R2 (FOXR2) degradation in lung cancer cells,^[^ ^49^ ^]^ highlighting its multifaceted role in cell death through the ubiquitination and degradation of key proteins.

PARK7 protects against oxidative stress and maintains mitochondrial integrity, key factors in Parkinson's disease.^[^ ^50^ ^]^ In HEK293 cells, it suppresses ferroptosis by detoxifying MGO and limiting protein glycation and lipid peroxidation.^[^ ^26^ ^]^ In contrast, our study shows that GLO1 dysfunction—but not PARK7 deficiency—promotes ferroptosis via MGO accumulation in cancer cells, with PJA1‐mediated GLO1 degradation acting as an upstream regulatory mechanism. These findings underscore MGO homeostasis as a critical determinant of ferroptosis sensitivity, with PARK7 and GLO1 functioning in a cell type–dependent manner to limit MGO toxicity.

We also reveal the role of MGO in inducing autophagy to accelerate ferroptosis. Autophagy is an important defense mechanism that promotes survival during energy stress, such as starvation. However, excessive or selective autophagy targeting specific substrates can mediate cell death, a process known as autophagy‐dependent cell death.^[^ ^51^ ^]^ Previous studies have shown that transmembrane protein 164 (TMEM164) selectively mediates ATG5‐dependent autophagosome formation during ferroptosis, rather than during starvation.^[^ ^52^ ^]^ Additionally, the selective degradation of ferritin, lipid droplets, solute carrier family 40 member 1 (SLC40A1, also known as ferroportin‐1), and GPX4 enhances ferroptosis sensitivity in a context‐dependent manner.^[^ ^53^, ^54^, ^55^, ^56^ ^]^ Our study demonstrates that MGO induces AMPK‐ and ATG‐dependent autophagy, which selectively degrades ferritin and GPX4, leading to iron‐dependent oxidative damage and ferroptosis. Further investigation is required to elucidate the specific checkpoint mechanisms through which MGO induces both pro‐survival and pro‐cell death autophagy.^[^ ^23^ ^]^

We also show that genetic or pharmacological inhibition of the GLO1 pathway enhances ferroptosis‐mediated tumor suppression in PDAC, a challenging solid cancer with poor survival rates and limited treatment options.^[^ ^57^ ^]^ Although several compounds can induce ferroptosis in vitro, few have demonstrated efficacy in vivo due to their short half‐life.^[^ ^58^ ^]^ IKE exhibited improved pharmacokinetics in vivo and was used to test the anticancer activity of ferroptosis in preclinical models.^[^ ^42^ ^]^ We found that *PJA1* knockout inhibited, whereas *PJA1* overexpression enhanced, the anticancer activity of IKE in xenograft models. In immunocompetent mouse models, the combination of a GLO1 inhibitor and IKE enhanced the cytotoxic T cell response, which is crucial for antitumor immunity. The observation that low GLO1 expression in human PDAC tumor tissue correlates with increased sensitivity to IKE suggests that assessing GLO1 expression may help identify patients suitable for ferroptosis‐targeted therapy. Moreover, we observed an inverse relationship between PJA1 expression and GLO1 levels with patient survival, further supporting the hypothesis that PJA1‐dependent GLO1 degradation contributes to tumor suppression.

Although our findings are strongly supported by preclinical models, further clinical validation is warranted. Given the considerable heterogeneity of PDAC, studies involving larger patient cohorts—with comprehensive clinical annotation and longitudinal follow‐up—will be essential to establish the prognostic and predictive significance of PJA1 and GLO1 expression. The integration of clinical genomics with tissue‐based validation will be critical for translating these insights into personalized therapeutic strategies.

In summary, our study uncovers a previously unrecognized metabolic regulatory pathway in ferroptosis. Targeting the PJA1–GLO1 axis to enhance MGO accumulation represents a promising approach to sensitize tumor cells to ferroptosis‐based therapies.

## 4 Experimental Section

### Reagents

MG132 (S2619), tetrathiomolybdate (E1166), liproxstatin‐1 (S7699), hydroxychloroquine (S4430), Z‐VAD‐FMK (S7023), necrosulfonamide (S8251), erastin (S7242), RSL3 (S8155), CCT137690 (S2744), staurosporine (S1421), SBI‐0206965 (S7885), gemcitabine (S1714), nab‐paclitaxel (E1068), Torin1 (S2827), and elesclomol‐Cu (E4801) were obtained from Selleck Chemicals. BrBzGCp2 (HY‐136684) was sourced from MedChemExpress. MGO (ICN15555825) was purchased from Thermo Fisher Scientific. Antibodies against MAP1LC3B (catalog numbers 2775 and 3868), ATG5 (12994), ATG7 (2631), GPX4 (52455), FTH1 (4393), AIFM2 (24972), SLC7A11 (12691), AMPK (2532), p‐AMPK (2535), K48‐linkage‐specific polyubiquitin (8081), K63‐linkage‐specific polyubiquitin (5621), CD8 (98941), CD4 (25229), SQSTM1 (5114), and ACTB (4970) were obtained from Cell Signaling Technology. Antibodies against GLO1 (MA1‐13029), GLO2 (17196‐1‐AP), CUL7 (13738‐1‐AP), and PJA1 (17687‐1‐AP) were purchased from Thermo Fisher Scientific.

### Cell Culture

PANC1 (CRL‐1469), HCT116 (CCL‐247), HT1080 (CCL‐121), and BxPC‐3 (CRL‐1687) cell lines were sourced from the American Type Culture Collection (ATCC). The KPC mouse cell line was kindly provided by Dr. David Tuveson. The inducible *Gpx4^−/−^* Pfa1 cells were generously provided by Dr. Marcus Conrad. All cell lines were cultured in Dulbecco's modified Eagle's medium (DMEM; Thermo Fisher Scientific, 11885084) supplemented with 10% heat‐inactivated fetal bovine serum (FBS; Millipore, TMS‐013‐B) and 1% penicillin‐streptomycin solution (Thermo Fisher Scientific, 15070‐063). The cultures were maintained at 37 °C in a humidified incubator with 5% CO_2_. Mycoplasma contamination was regularly tested, and all cell lines were authenticated using CLA IdentiFiler™ Plus PCR Amplification Kit (Thermo Fisher Scientific, A47624).

For compound preparation, dimethyl sulfoxide (DMSO; Sigma‐Aldrich, S‐002‐M) was used to prepare stock solutions. The DMSO concentration in the final working solutions was kept below 0.01%, and the same concentration was used as the vehicle control in all cell culture assays.

### Animal Experiments

All animal care and experimental procedures were conducted in accordance with the guidelines of the Association for Assessment and Accreditation of Laboratory Animal Care (AAALAC; http://www.aaalac.org) and the ARRIVE guidelines (https://arriveguidelines.org/). All protocols were approved by the Institutional Animal Care and Use Committees of Guangzhou Medical University (Approval No. S2023‐786) and Jilin University (Approval No. 2022071). Experimental and control groups of animals were matched for sex and age, with no exclusions made at the time of harvest. The sample size for animal studies was not determined using statistical methods, and investigators were not blinded to group allocation or outcome assessment.

For the induction of murine subcutaneous tumors, 5 × 10^6^ PANC1 or HCT116 cells were injected into the flanks of 6–8 week old female NSG (Jackson Laboratory, 0005577) mice. After 10 days post‐inoculation, treatment regimens were initiated, which included daily intraperitoneal (i.p.) administration of either a vehicle control or IKE (30 mg kg^−1^) for 5 days per week over a 3‐week period (n = 5 mice per group). Tumors were measured weekly, and tumor volumes were calculated using the formula: volume = length × width^2^ × π/6. In addition, patient‐derived organoids from liver metastases of PDAC patients were generated and subsequently expanded as PDXs in NSG mice.^[^ ^59^, ^60^, ^61^, ^62^ ^]^ The use of patient samples was obtained through written informed consent and approved by the Institutional Review Board of Jilin University (2024020708).

To establish orthotopic tumors, 5 × 10^5^ KPC cells were surgically implanted into the tail of the pancreas of 6–8 week‐old C57BL/6J mice (male:female = 1:1, n = 20 mice per group).^[^ ^33^, ^63^ ^]^ Ten days following implantation, the mice were randomly assigned to treatment groups and received either a vehicle control or IKE (30 mg kg^−1^, i.p., once daily, 5 days per week) for a duration of 3 weeks. Additionally, the treatment groups were administered BrBzGCp2 (50 mg kg^−1^, i.p., once daily, 5 days per week) as specified. Animal survival was monitored weekly. The doses of IKE and BrBzGCp2 were selected based on prior studies.^[^ ^42^, ^64^ ^]^

### Western Blot

Whole cells were lysed three times using cell lysis buffer (Cell Signaling Technology, 9803), supplemented with a protease inhibitor cocktail (Cell Signaling Technology, 5871). Each lysis step was performed on ice for 10 min. Protein concentrations were determined using a bicinchoninic acid assay (Thermo Fisher Scientific), and protein (20–30 µg) from each sample was loaded onto 4%‐12% Criterion XT Bis‐Tris gels (Bio‐Rad). Electrophoresis was carried out in XT MES running buffer (Bio‐Rad), followed by transfer to polyvinylidene difluoride membranes (Bio‐Rad) using the Trans‐Blot Turbo Transfer Pack and System (Bio‐Rad). Membranes were blocked for 1 h with Tris‐buffered saline with Tween 20 (Cell Signaling Technology) containing 5% skim milk (Cell Signaling Technology, 9999). The membranes were then incubated overnight at 4 °C with primary antibodies (1:1000 dilution). After three washes with TBST, the membranes were incubated with goat anti‐rabbit or anti‐mouse IgG horseradish peroxidase‐conjugated secondary antibodies (1:1000; Cell Signaling Technology, 7074 or 7076) at room temperature for 1 h, followed by additional washes. Chemiluminescent detection was performed using the SuperSignal West Pico Chemiluminescent Substrate (Thermo Fisher Scientific, 34580), and the blots were visualized using the ChemiDoc Touch Imaging System (Bio‐Rad). The images were then analyzed with Image Lab Software (Bio‐Rad).

### Cytotoxicity Assays

To assess cell viability, the Cell Counting Kit‐8 (CCK‐8) assay was used (Dojindo Laboratories, CK04), which is based on the reduction of water‐soluble tetrazolium salt (WST‐8) to form an orange formazan product. Cells were seeded in 96‐well plates and allowed to adhere and grow under the desired experimental conditions. Following treatment, the CCK‐8 reagent was added to each well, and the cells were incubated for 2 h. The reagent was metabolized by viable cells, resulting in the production of formazan, which was quantified by measuring the absorbance at 450 nm using a microplate reader (TECAN).

To assess cell death, trypan blue staining was used (Thermo Fisher Scientific, 15250061). Trypan blue is a vital dye that is impermeable to live cells with intact membranes but enters dead or dying cells with compromised membranes, staining them blue. To perform the assay, cells were first harvested and resuspended in medium. An equal volume of 0.4% trypan blue solution was then added to the cell suspension. The mixture was incubated for 2–5 min at room temperature, allowing the dye to penetrate the dead cells. After incubation, a small aliquot of the cell suspension was placed on a hemocytometer, and the cells were examined under a light microscope.

### qPCR

Total RNA was extracted and purified from samples using the RNeasy Plus Mini Kit (QIAGEN, 74136). First‐strand cDNA synthesis was then carried out from RNA (1 µg) using the iScript cDNA Synthesis Kit (Bio‐Rad, 1708890). The reaction (20 µl) mixtures were prepared by combining iScript Select reaction mix (4 µl), gene‐specific enhancer solution (2 µl), reverse transcriptase (1 µl), the gene‐specific assay pool (1 µl, 20×, 2 mm), and RNA diluted in RNase‐free water (12 µl). The cDNA generated from the samples was subsequently amplified by qPCR using the following specific primers: *GLO1* (CACTCTACTTCTTGGCTTATGAGG and GGGTCTCATCATCTTCAGTGCC), *GPX4* (ACAAGAACGGCTGCGTGGTGAA and GCCACACACTTGTGGAGCTAGA), *FTH1* (TGAAGCTGCAGAACCAACGAGG and GCACACTCCATTGCATTCAGCC), and *RNA18S RNA* (ACCCGTTGAACCCCATTCGTGA and GCCTCACTAAACCATCCAATCGG). The amplification was conducted using the CFX96 Touch Real‐Time PCR Detection System (Bio‐Rad), with data analysis performed using the CFX Manager software (Bio‐Rad). The results were normalized to *RNA18S RNA*, and fold change was calculated using the 2^‐ΔΔCt^ method. mRNA concentrations were expressed in arbitrary units, with the untreated group designated as a baseline value of 1.

### Gene Editing and Mutation

Pre‐designed guide RNAs (gRNAs) targeting the human genes *ATG5* (ATCACAAGCAACTCTGGAT; HSPD0000055154), *ATG7* (CTAGCTACATTGCAACCCA; HSPD0000063209), *CUL7* (AGACCTATTGGGAGTCCAA; HSPD0000057844), *PJA1* (TGTAGCGAATATGTGAAGG; HSPD0000107166), *GLO1* (HSPD0000016404; ATGGCAATTCAGACCCTCG) and *PARK7* (ATGTGGTGGTTCTACCAGG; HSPD0000068522) were obtained from Sigma‐Aldrich. Gene editing was performed using the CRISPR‐Cas9 technique, following the protocol established by Dr. Feng Zhang's laboratory at the Massachusetts Institute of Technology.^[^ ^65^ ^]^ After transduction, the cells were selected using puromycin (5 µg ml^−1^; InVivoGen, ant‐pr‐1) for 5 days, with the puromycin concentration determined based on cell killing curves.

To generate the human PJA1 C598A, GLO1 K47R/K48R, and GLO1 K157R/K159R mutations, a site‐directed mutagenesis approach was employed. Plasmids containing the wild‐type *PJA1* (OriGene, RC224490) and *GLO1* (OriGene, RC203826) coding sequences served as templates for the mutagenesis reactions. For the *PJA1* C598A mutation, primers were designed to substitute the cysteine codon (TGC) at position 598 with an alanine codon (GCC). The primers used were: Forward: 5’‐TGTGCTGAGGAGGGTGCGGCCGAGGTTTGGGAGGTT‐3’; Reverse: 5’‐ACCTCCCAAACTCCGGCGGCAGTGACGGTGA‐3’. For the *GLO1* K47R/K48R double mutation, the following primers were used: Forward: 5’‐CTGGTGGCGGACAGAAGGGAGGAGAAG‐3’; Reverse: 5’‐CTTCTCCTCCCTCCTTCTGCCGCCACCAG‐3’. For the *GLO1* K157R/K159R double mutation, the following primers were used: Forward: 5’‐TTCTCCTGGGTCAGGGTCAGGAAGGAGTTC‐3’; Reverse: 5’‐GAACTCCTTCCTCCTGACCCTGACCCAGGAGA‐3’. Mutations were introduced via PCR, and resulting clones were screened by Sanger sequencing to confirm the presence of the intended mutations.

### Immunoprecipitation

Cell lysates were prepared on ice using ice‐cold radioimmunoprecipitation assay (RIPA) buffer containing Tris‐HCl (pH 7.5, 20 mm), NaCl (150 mm), Na_2_EDTA (1 mm), EGTA (1 mm), 1% NP‐40, 1% sodium deoxycholate, sodium pyrophosphate (2.5 mm), β‐glycerophosphate (1 mm), Na_3_VO_4_ (1 mm), leupeptin (1 µg ml^−1^), and phenylmethylsulfonyl fluoride (PMSF, 1 mm) (Cell Signaling Technology, 9806). Cells were incubated in the lysis buffer for 5–10 min, followed by centrifugation at 14 000 x g for 10 min at 4 °C to remove cell debris. Protein concentrations in the resulting supernatant were determined using the BCA protein assay. For immunoprecipitation, equal amounts of protein were pre‐cleared by incubating with protein A/G sepharose beads (Abcam, ab193262) for 3 h at 4 °C. After pre‐clearing, the samples were incubated overnight at 4 °C with either irrelevant IgG or specific antibodies (2–3 µg ml^−1^), along with protein A/G sepharose beads, with gentle agitation. Following incubation, the beads were washed thoroughly with phosphate‐buffered saline (PBS; Cell Signaling Technology, 9808). The bound proteins were then eluted by boiling the beads in 2× SDS sample buffer, followed by analysis using SDS‐polyacrylamide gel electrophoresis.

### Immunohistochemistry

Formalin‐fixed, paraffin‐embedded tumor tissue sections (5 µm thick) were processed following standard immunohistochemistry protocols. Briefly, the slides were deparaffinized with xylene and rehydrated through a series of ethanol concentrations in water. Antigen retrieval was performed by incubating the slides in 10 mm sodium citrate buffer (pH 6.0) preheated in a microwave for 2 min, followed by incubation at 95–98 °C for 10 min, then cooling to room temperature for 30 min. Endogenous peroxidases were inactivated by treating the slides with 3% hydrogen peroxide for 10 min. After two 5‐min washes with PBS, endogenous avidin and biotin were blocked using 1× Animal‐Free Blocking Solution (Cell Signaling Technology, 15019). Following an additional two 5‐min PBS washes, primary antibodies (1:100‐1:200) were applied and incubated overnight at 4 °C in a humidified chamber. After rinsing with PBS, slides were incubated with 1‐3 drops of SignalStain Boost IHC Detection Reagent (Cell Signaling Technology, 8114) for 30 min at room temperature. After washing three times with PBS, the slides were treated with SignalStain DAB (Cell Signaling Technology, 8059) for 3–5 min. Sections were then washed with distilled water, counterstained with hematoxylin (Cell Signaling Technology, 14166), dehydrated through ethanol and xylene, and mounted using SignalStain Mounting Medium (Cell Signaling Technology, 14177). Images were captured and quantified from five fields using an EVOS microscope (Thermo Fisher Scientific).

### ELISA and Biochemical Assay

The concentrations of TNF (Thermo Fisher Scientific, 88‐7324‐22), IFNG (Thermo Fisher Scientific, A41150), IL2 (Thermo Fisher Scientific, A35603), MDA (Sigma‐Aldrich, MAK568), Fe^2+^ (Thermo Fisher Scientific, EEA009), ATP ( Thermo Fisher Scientific, A22066), and MGO (abbexa, abx257293) in the indicated samples were quantified using ELISA, following the manufacturers' protocols.

### Protein Stability Assay

To assess protein stability, cells were treated with erastin in the presence of cycloheximide (100 µg mL^−1^) to inhibit *de novo* protein synthesis and were harvested at the indicated time points. Whole‐cell lysates were prepared using RIPA buffer supplemented with protease inhibitors, and equal amounts of protein were analyzed by SDS‐PAGE followed by immunoblotting with specific antibodies.

### Autophagic Flux Analysis

Autophagic flux was evaluated using a tandem fluorescent probe, GFP‐MAP1LC3‐RFP‐MAP1LC3ΔG,^[^ ^36^, ^66^ ^]^ stably expressed in PANC1 cells. Cells were plated in 96‐well plates at a density of 1 × 10⁴ cells per well and treated with MGO for 24 h in triplicate. GFP and RFP fluorescence intensities were measured using a BioTek Cytation 5 microplate reader. Excitation/emission wavelengths were set at 485/528 nm for GFP and 555/584 nm for RFP.

### Transmission Electron Microscopy Analysis

To assess ultrastructural features associated with ferroptosis, cells were fixed in 2.5% glutaraldehyde in phosphate buffer (pH 7.4, 0.1 M) at 4 °C overnight, post‐fixed with 1% osmium tetroxide, dehydrated through a graded ethanol series, and embedded in epoxy resin.^[^ ^67^, ^68^ ^]^ Ultrathin sections (70–90 nm) were stained with uranyl acetate and lead citrate, and examined using a transmission electron microscope (e.g., JEOL JEM‐1400) at 80 kV. Characteristic ferroptotic changes—including increased membrane density, condensation of mitochondrial membranes, and loss or reduction of mitochondrial cristae—were consistently observed in treated cells compared to controls. Representative images were captured digitally for morphological comparison.

### Statistical Analysis

All experiments were performed with at least three independent biological replicates. Data are presented as means ± SD, as indicated in the figure legends. Data collection and analysis were performed using GraphPad Prism 8.4.3 software. Unpaired Student's t‐tests were employed to compare the means of two groups, while one or two‐way analysis of variance (ANOVA) with Tukey's multiple comparisons test was utilized for comparisons among multiple groups. Differences in mortality rates between groups were assessed using log‐rank tests. Statistical significance was defined as a p value of <0.05.

## Conflict of Interest

The authors declare no conflict of interest.

## Author Contributions

X.Z., L.H., Z.W., H.Y., J.L., Z.L., X.D., and E.D. performed the experiments and analyzed the data. D.T., Z.L., X.D., and E.D. designed the study and wrote the manuscript. R.K. assisted in data interpretation and edited the manuscript. All authors read and approved the final manuscript.

## Supporting information



Supporting Information

## Data Availability

The data that support the findings of this study are available from the corresponding author upon reasonable request.
