# Supplementary material for: Targeting Methylglyoxal Metabolism to Enhance Ferroptosis Sensitivity in Tumor Therapy
Source: Adv Sci (Weinh). 2025 Sep 4;12(40):e05356. doi: 10.1002/advs.202505356 (PMC12561271; doi:10.1002/advs.202505356)
Supplement: Supplementary file 1 — Supporting Information [file ADVS-12-e05356-s001.docx]

**Table S1. Ubiquitination score of each lysine in human GLO1**

| Position | Score | Explanation |
| --- | --- | --- |
| K27 | 1 | Near N-terminus |
| K44 | 1 | Near other lysines (K47/K48) |
| K47 | 2 | Near N-terminus + clustered |
| K48 | 2 | Near N-terminus + clustered |
| K66 | 0 | Internal, isolated |
| K72 | 0 | Internal, isolated |
| K85 | 0 | Internal, isolated |
| K90 | 1 | Clustered with K91 |
| K91 | 1 | Clustered with K90 |
| K98 | 0 | Internal, isolated |
| K106 | 0 | Internal, isolated |
| K113 | 0 | Internal, isolated |
| K122 | 0 | Internal, isolated |
| K138 | 0 | Internal, isolated |
| K143 | 0 | Internal, isolated |
| K144 | 0 | Internal, isolated |
| K157 | 2 | Near C-terminus + clustered |
| K159 | 2 | Near C-terminus + clustered |
